# Clinical characteristics of free flaps for oral and maxillofacial reconstruction: a retrospective study of 700 flaps over 3 years

**DOI:** 10.7717/peerj.21245

**Published:** 2026-06-09

**Authors:** Shuai Li, YanPing Xie, JiaLi Xie, Yi Wei, GuoSheng Chen, YiXing Li, QingTiao Xie, JiaXiao Wu

**Affiliations:** 1 Guangxi Key Laboratory of Oral and Maxillofacial Rehabilitation and Reconstruction, Naning, Guangxi, China; 2 Guangxi Clinical Research Center for Craniofacial Deformity, Nanning, Guangxi, China; 3 Department of Oral and Maxillofacial Surgery, College of Stomatology, Guangxi Medical University, Nanning, Guangxi, China; 4 Department of Pediatric Dentistry, College of Stomatology, Guangxi Medical University, Nanning, Guangxi, China

**Keywords:** Flap crisis, Oral and maxillofacial reconstruction, Explore, Free flap, Flap failure

## Abstract

**Objective:**

This study investigated the clinical characteristics and management of free flaps for oral and maxillofacial reconstruction.

**Methods:**

A total of 689 subjects were categorized into the no-flap crisis group (620 flaps in 620 subjects) and the flap crisis group (80 flaps in 69 subjects). Demographic and clinical data, including gender, age, disease types, body mass index, preoperative chemoradiotherapy, poor lifestyle habits, postoperative length of stay, medical cost, intraoperative blood loss, during of surgery, hypertension and diabetes mellitus, were analyzed. In the flap crisis group, the distribution characteristics of flap types and the timing of crisis were examined.

**Results:**

The overall survival rate of free flaps was 95% (95% CI [93.4–96.6]), with a salvage rate of 49.28% (95% CI [37.2–61.4]). The two groups did not significantly differ in age, body mass index, preoperative chemoradiotherapy, hypertension, diabetes mellitus or poor lifestyle habits. However, significant differences were observed in postoperative length of stay, during of surgery, medical cost and intraoperative blood loss. Within the flap crisis group, 65 flaps (94.20%) underwent crisis within the first 84 hours, and flap crisis showed a strong association with flap failure.

**Conclusion:**

Free flap reconstruction is a dependable method, with most crises occurring within 84 hours, highlighting the imperative for early surgical exploration to avert necrosis.

## Introduction

Over the past few decades, free flap transfer has become a cornerstone of oral and maxillofacial reconstruction. The survival rate of these flaps is generally considered to be in the region of 95% (Walia et al., 2022). Free flaps cover defects and restore partial functions in the oral and maxillofacial regions, improving swallowing and facial appearance. Successful transplantation not only boosts the confidence of both doctors and patients but also helps reduce medical cost. However, flap failure may lead to functional and cosmetic impairments, additional surgery, prolonged hospitalization, increased medical expenses, and delays in adjuvant therapy (Eskander et al., 2018).

Close postoperative monitor is essential for assessing the perfusion of free flaps. Critical parameters include flap color, temperature, capillary refill, turgor, and bleeding characteristics (Megias Barrera et al., 2019). Compromised perfusion triggers progressive changes in these indicators. Without timely intervention, the transplanted flap is at risk of failure. Currently, the optimal time window for treating flap crisis remains unclear. Animal models of venous congestion show changes in the color, texture, and histology of skin flaps over time suggesting that the time window for thrombogenesis is approximately 4 hours (Du et al., 2015). However, such findings may not directly translate to clinical practice. Even when a flap crisis is suspected, there is no consensus regarding the most effective approach, whether observation, drug treatment or reoperation (Wang et al., 2020).

Furthermore, the characteristics of flap types and flap crises require further research (Yang et al., 2016). Although various methods exist to manage irreversible flap compromise, many aspects of these strategies still need further optimization and clarification (Walia et al., 2022).

This study analyzed clinical characteristics of free flaps used in oral and maxillofacial reconstruction over the past 3 years.

## Patients and Methods

A retrospective analysis was conducted on a consecutive series of 700 free flaps performed for oral and maxillofacial reconstruction in 689 subjects at College of Stomatology, Guangxi Medical University in Nanning, China. The study included flaps and related data of subjects from the Department of Oral and Maxillofacial Surgery between December 15, 2020, and September 20, 2023.

The study was undertaken with the principles of the Declaration of Helsinki. Given the retrospective design of the study, the requirement for informed consent was waived. Ethical approval was obtained from the Ethics Committee of Stomatology Hospital Affiliated with Guangxi Medical University (Approval No: 2024003).

The inclusion criteria as follows: (1) subjects diagnosed with tumors; (2) subjects who underwent major resections and presented with defects in oral and maxillofacial regions; (3) subjects treated with free flap reconstruction including anterolateral thigh flap (ALTF), radial forearm free flap (RFFF), deep circumflex iliac artery free flap (DCIA) or fibula flap, and flap types were decided by the characteristics of recipient defects and donor sites; (4) All procedures were performed by surgeons with more than 5 years of clinical experience.

Postoperative flap perfusion was evaluated through bedside observation, focusing on key indicators such as flap color, temperature, capillary refill, turgor, and bleeding. Postoperative monitoring was performed following a tapering frequency schedule: every 30 min on day 1, hourly on day 2, and every 4 h from days 3 to 7. Additionally, arterial flow was evaluated three times daily using a needle prick test. The criteria for identification of flap crisis and flap failure were as follows:

1. Venous crisis (VC): Appearance of dark spots or violet patches worsening within 30 min.
2. Arterial crisis (AC): Skin wrinkling and pallor, which was confirmed by Doppler ultrasound or subcutaneous puncture.
3. Mixed crisis (MC): A mixed pattern of vascular crisis exhibiting characteristics of both arterial and venous compromise.
4. Flap failure: Pale, bloodless puncture, wound dehiscence, odor, or infection.
5. All flap crises and failures were confirmed by two surgeons, each with more than five years of clinical experience. Completed necrosis of flap was defined as flap failure.

The subjects were categorized into the flap crisis group (80 flaps in 69 subjects) and the no flap crisis group (620 flaps in 620 subjects). The clinical and demographic information of subjects was recorded, including gender, age, body mass index (BMI), disease types, preoperative chemoradiotherapy, poor lifestyle habits (smoking or alcohol use), postoperative length of hospital stay, medical cost, intraoperative blood loss, during of surgery, hypertension, diabetes mellitus (DM) and the timing and type of flap crisis.

### Statistical analysis

The data were analyzed using descriptive statistics, including means and standard deviations. Chi-square test, t test, multivariable logistic regression analysis and generalized linear mixed model analysis were applied. Patient ID was included as a random effect, and flap count—accounting for both multiple and salvage flaps—was incorporated as a model parameter. Analyses were conducted using SPSS software (version 23; IBM Corp., Armonk, NY, USA). A *p*-value of <0.05 was considered statistically significant.

## Results

A total of 689 subjects underwent oral and maxillofacial reconstruction between December 15, 2020, and September 20, 2023, involving 700 free flaps. The overall viability rate of the 700 flaps was 95% (*n* = 665, 95% CI [93.4–96.6]). The distribution of flap types was as follows: ALTF 75.43% (*n* = 528), Fibula 17.57% (*n* = 123), RFFF 5.86% (*n* = 41) and DCIA 1.14% (*n* = 8) (Table 1).

The characteristics of the subjects are summarized in Table 2. The disease types in both groups included cancers of the tongue, gum, cheek, and floor of the mouth, as well as ameloblastoma, bony tumor, and mucoepidermoid carcinoma. The mean age was 52.31 years in the no-flap crisis group and 50.87 years in the flap crisis group (*p* = 0.398). No significant differences were detected in the prevalence of poor lifestyle habits (213 *vs.* 28, *p* = 0.352) or preoperative chemoradiotherapy (53 *vs.* 8, *p* = 0.374). The no-flap crisis group had a higher prevalence of both DM (61 *vs.* 10, *p* = 0.442) and hypertension (104 *vs.* 16, *p* = 0.341) compared to the flap crisis group. The mean BMI was higher in the no-flap crisis group than in the flap crisis group (22.52 *vs.* 21.92; *p* = 0.170).

**Table 1:** Distribution of flap crisis and flap failure.

Type of flap	Cases of all	Flaps crisis	Flap failure	Failure proportion in different flap types
RFFF	41 (5.86%)	1 (1.45%)	1 (2.86%)	2.44% (1/41)
ALTF	528 (75.43%)	51 (73.91%)	26 (74.28%)	4.92% (26/528)
Fibula	123 (17.57%)	16 (23.19%)	7 (20.00%)	5.69% (7/123)
DCIA	8 (1.14%)	1 (1.45%)	1 (2.86%)	12.5% (1/8)
Total	700 (100%)	69 (100%)	35(100%)	

**Notes.**

ALTF, anterolateral thigh fiap; RFFF, radial forearm free flap; DCIA, deep circumfiex iliac artery free flap.

**Table 2:** Demographic of subjects.

Factors	Non flap crisis group *n* = 620	Flap crisis group *n* = 69	Univariate analysis
Gender	Male: 434 (70%)	Male: 50 (72.46%)	–
Years (years old)	52.31 ± 13.44	50.87 ± 13.57	0.398
Poor lifestyle habits	213	28	0.352
Preoperative chemoradiotherapy	53	8	0.374
DM	61	10	0.442
Hypertension	104	16	0.341
Postoperative length of stay (days)	13.82 ± 5.90	19.84 ± 8.64	0.000
Intraoperative blood loss (mL)	598.85 ± 355.17	691.88 ± 500.32	0.049
BMI (kg/m^2^)	22.52 ± 3.44	21.92 ± 3.44	0.170
Medical cost (CNY)	51,271.48 ± 12,496.88	72,484.24 ± 44,748.17	0.000
During of surgery (hours)	7.33 ± 1.89	7.98 ± 1.77	0.007
Disease types			
TC	220	24	
Gum cancer	72	11	
Buccal cancer	88	6	
FOMC	83	7	
AM	39	4	
Bony tumor	112	16	
MEC	6	1	
Total	620	69	

**Notes.**

CNY, Chinese Yuan; TC, Tongue cancer; FOMC, Floor of mouth cancer; AM, Ameloblastoma; MEC, mucoepidermoid carcinoma.

Subjects experiencing flap crisis demonstrated significantly greater intraoperative blood loss (691.88 ml *vs.* 598.85 ml, *p* = 0.049), longer during of surgery (7.98 h *vs.* 7.33 h, *p* = 0.007) and extended hospital stays (19.84 days *vs.* 13.82 days, *p* = 0.000) compared to those without crisis. Consequently, total medical cost were significantly higher in the crisis group (72,484.24 CNY *vs.* 51,271.48 CNY, *p* = 0.000).

Multivariable logistic regression identified during of surgery as significantly associated with flap crisis after adjusting for potential confounders (*p* = 0.019, Tablse S1). A generalized linear mixed model was constructed assess factors associated with flap crisis. The model was fitted on data from 689 subjects and included the following candidate predictors: flap count, intraoperative blood loss, BMI, during of surgery, poor lifestyle habits, preoperative chemoradiotherapy, DM, and hypertension. Flap count exhibited a significant association with flap crisis (*p* = 0.000, Tablse S2).

The characteristics of flap crisis are summarized, detailing distribution of crisis types (Table 3 & Fig. S1) and flap types (Table 1) involved. The incidence of flap crisis was highest within the first 12 h, with 40 flaps (57.97%) occurring during this period. There were 13 flaps (18.84%) within 12 to 24 h and five flaps (7.25%) within 24 to 36 h. Overall, 58 flaps (84.06%) experienced flap crisis within the first 36 h. With each crisis case treated as an independent observation, Kaplan–Meier analysis of the 69 flap crisis cases revealed a decline in probability over time, decreasing to less than 5% by 84 h post-transplantation (Figs. S1 & S2). Furthermore, the number of sole VC was more than that of sole AC (57 cases *vs.* 8 cases, *p* = 0.023, Table 3). There were four cases involved MC.

**Table 3:** The time profile of flap crisis after free flap transfer and subsequent exploration outcomes.

Time distribution (h)	Without necrosis		Partial necrosis		Flap failure			Crisis proportion	*P* value
	VC	AC	VC	AC	VC	AC	MC		
<12 hours	13	1	6	0	13	5	2	57.97%	
12–24 hours	8	0	2	0	2	0	1	18.84%	
24–36 hours	2	0	0	0	2	1	0	7.25%	
36–48 hours	0	0	1	0	1	0	1	4.35%	
48–60 hours	0	0	0	0	2	0	0	2.90%	
60–72 hours	0	0	0	0	1	1	0	2.90%	
72–84 hours	1	0	0	0	0	0	0	1.45%	
84–96 hours	0	0	0	0	1	0	0	1.45%	
96–108 hours	0	0	0	0	1	0	0	1.45%	
108–120 hours	0	0	0	0	1	0	0	1.45%	
Total	24	1	9	0	24	7	4	100%	
VC: AC									0.023

**Notes.**

(1) Venous crisis, VC; Arterial crisis, AC; Mixed crisis, MC.

(2) Flap survival was defined to encompass cases of partial necrosis.

The incidence of flap failure was highest within the first 12 h (Table 3 & Fig. S1), with 20 flaps (57.14%) occurring in this timeframe, followed by 3 flaps (8.57%) between 12 and 24 h, and three flap (8.57%) between 24 and 36 h. Within the first 36 h, 26 flaps (74.29%) failed. The incidence of flap failure for ALTF, DCIA, fibula, and RFFF were 4.92%, 12.5%, 5.69%, and 2.44%, respectively (Table 1).

Flap salvage rate was further analyzed based on the timing of exploration after onset of flap crisis (Table 4). The salvage rates were 54.17% (13 of 24) within 4 h and 66.67% (8 of 12) from 4 to 10 h, respectively. 16 flaps underwent delayed exploration (four flaps surviving). In total, 35 flaps failed. Strategies were employed for these failed flaps, including free flaps (*n* = 11), adjacent flaps (*n* = 4), and debridement (20 cases) (Table 5). Postoperative length of stay (*p* = 0.002), intraoperative blood loss (*p* = 0.456), and medical cost (*p* = 0.350) increased for subjects with failed flaps in flap crisis group (Table 6).

**Table 4:** Explore results of flap crisis based on different time.

Explore time	Success (salvage rate)	Failure
<4 hours	13 (54.17%)	11 (45.83%)
4–10 hours	8 (66.67%)	4 (33.33%)
10–24 hours	9 (52.94%)	8 (47.06%)
Delayed explore (>24 hours)	4 (25.00%)	12 (75%)
Total	34 (49.28%)	35 (50.72%)

**Table 5:** Treatment strategies of failed flap.

Strategies	Failed number	Treatment strategies of failed flap				
Failed flaps		Debridement	ALTF	Fibula	RFFF	PMMF
RFFF	1	0	1	0	0	0
ALTF	26	14	6	0	2	4
Fibula	7	6	1	0	0	0
DCIA	1	0	0	1	0	0
Total	35	20	8	1	2	4

**Notes.**

Pectoralis major myocutaneous flap (PMMF).

**Table 6:** Flap failure related problems in flap crisis group.

	Flap crisis without failure *n* = 34	Flap crisis with failure, *n* = 35	*P* value
Postoperative length of stay (days)	16.65 ± 7.26	22.94 ± 8.84	0.002
Medical cost (CNY)	67,277.08 ± 56,044.33	77,542.62 ± 30,045.91	0.350
Intraoperative blood loss (mL)	645.88 ± 426.15	736.57 ± 565.86	0.456

## Discussion

Free flaps are widely used in the reconstruction of oral and maxillofacial defects, offering high success rates, favorable aesthetic outcomes, and low comorbidity (Russell, Pateman & Batstone, 2021). Despite these advantages, postoperative flap crises still occur (Chorath et al., 2021), with reported rates ranging from 2.8% to 12.4% (Classen & Ward, 2006; Devine et al., 2001; Nakatsuka et al., 2003). To minimize the risk of flap failure, early detection and prompt treatment of flap crisis are recommended to achieve favorable outcomes, as salvage rate for compromised flaps ranged from 16.7% to 75% (Harashina, 1988; Irons, Wood & Schmitt 3rd, 1987; Shen et al., 2021; Tsai et al., 1988). Although flap failure may lead to devastating or even fatal consequences, flap crisis can often be partially reversed with early detection and timely treatment (Shen et al., 2021). The time window for salvaging flap crisis distributed timing and flap type of flap crisis required further investigation (Yang et al., 2016). Moreover, there is a lack of consensus on the optimal methods for dealing with flap crisis (Wang et al., 2020; Chen et al., 2007).

In this study, the overall rate of free flap survival was 95% (95% CI [93.4–96.6]), while the flap crisis rate was 9.86% (69/700). With close monitoring and timely intervention, the salvage rate following exploration was 49.28% (95% CI [37.2–61.4]), consistent with the average outcomes reported worldwide (Largo et al., 2018).

Comparison of clinical features between the two groups showed that intraoperative blood loss and the during of surgery were associated with flap crisis. While multivariable logistic regression analysis revealed that the during of surgery acted as an independent factor (Table S1). Our results are consistent with the findings of Karolina Persson (etal Persson et al., 2025). Furthermore, generalized linear mixed model analysis demonstrated that flap count exhibited a significant association with flap crisis (Table S2).

The treatment of flap crisis resulted in a longer postoperative hospital stay and increased medical cost, as shown in Table 2. Previous research have identified the first 72 h as a critical time period for monitoring flap perfusion (Chen et al., 2007). This study found that nearly 95% of flap crises occurred within the first 84 h. During the initial 36 h, 26 flaps failed (74.29%). The correlation between flap crisis and flap failure was strong (*r* = 0.9763, *p* < 0.05, Fig. S1). Therefore, reducing the incidence of flap crisis can significantly decrease the flap failure rate. While previous studies have noted that flap crises and failures tend to occur early, this is the first study to describe their direct correlation (Yang et al., 2016).

This study used four types of free flaps, namely ALTF, fibula, RFFF and DCIA, for oral and maxillofacial reconstruction, with varying rates of flap crisis and failure for each flap type (Table 1). RFFF appeared to be the safest showing a lower rate of flap failure, likely due to its unique perfusion and reliable anatomy (Hekner, Roeling & Van Cann, 2016). Although ALTF is more commonly used for soft tissue reconstruction than RFFF, the flap crisis and failure rates were higher. This may be attributed to anatomical variations and the unique blood supply of the skin island. Previous study found that perforator flaps have a significantly higher risk of failure than those with multiple perforators (Chang et al., 2016). Despite these limitations, ALTF remains widely used because it offers ample tissue volume, minimal donor-site morbidity and well-concealed scarring, making it highly valuable for clinical reconstruction(Ranganath et al., 2022).

To assess the perfusion of the free flap, clinical monitoring was conducted following a tapering frequency schedule. In the flap crisis group, 69 flaps underwent exploration, of which16 flaps explored at a delayed stage (Table 4). Within 4 h of identifying the flap crisis, 24 flaps were explored, of which 13 survived. Between 4 and 10 h, 12 flaps were explored, with 8 surviving. Although our team unable to determine the precise interval between thrombosis formation and the onset of flap crisis, severe thrombosis formation was observed after more than 4 h. Based on the evidence from animal model and our clinical experience, it is recommended that exploration be performed as soon as possible, with the time interval ideally kept within 4 hours (Du et al., 2015).

During the study, the salvage rate for VC was significantly higher than that for AC (*p* = 0.023). Compared to VC, the timely detection of AC is more challenging. Recent research has made substantial progress in improving the early identification of flap compromise, and several novel monitoring devices have demonstrated promising effectiveness (Xie et al., 2023a; Xie et al., 2023b). However, their clinical implementation remains limited due to ethical considerations and financial constraints.

At our center, a period of more than 7 days post-transplantation is considered the threshold for determining the success of a free flap (Table 3). In this study, no flap failures were observed beyond this timeframe. Although cases of flap failure occurring after hospital discharge beyond 7 days post-transplantation, the incidence remains very low (Largo et al., 2018). Therefore, the first 7 days following flap transplantation may serve as a critical period for evaluating flap viability and predicting the likelihood of survival.

Despite prompt surgical exploration, some flaps, whether affected by AC or VC, became irreversibly compromised (Table 3). Salvaging these flaps posed a significant challenge and involved considerable risk (Wang et al., 2020). Hirudotherapy has been effectively used to treat venous compromise in free or pedicled flaps, with reported success rates ranging from 65% to 85%. Monitoring hemoglobin levels and prophylactic antibiotics are essential during leech therapy to ensure patient safety and treatment efficacy (Wang et al., 2020). In our earlier study, one case of the VC was treated solely with hirudotherapy resulting in the successful preservation of most of the flap.

Moreover, failed flaps were treated using free flap, adjacent flaps, or debridement (Table 5). These interventions were associated with a significant increase in postoperative hospital stay, intraoperative blood loss, and overall medical cost (Table 6). Postoperative flap perfusion relied on the pedicle vessels and revascularization from surrounding tissues. Previous studies have indicated that early perfusion of free flaps depends entirely on the pedicle, with the transition to tissue-based revascularization occurring between 6 days and 2 weeks postoperatively (Granzow et al., 2015). A 2-week interval is generally accepted as the consensus standard (Wang et al., 2020). Even if the pedicle vessels are compromised, the free flap may still receive adequate perfusion from the surrounding local tissue.

All subjects in this study maintained a specific head position for 5 to 7 days postoperatively, depending on the length of the vascular pedicle, the anastomosis site and the angle of the vessels (Wang et al., 2020). Frequent movement of the head can disrupt the stability of blood flow. Although thrombosis at the anastomosis site is the most common cause of flap failure, anticoagulation therapy increases the risk of bleeding and hematoma without reducing the likelihood of flap failure (Dawoud et al., 2022). No anticoagulant therapy was employed throughout the postoperative course. Vasospasm is additional complication that can lead to partial or complete flap loss. Various factors are considered potential triggers for vasospasm. Therefore, preventive or therapeutic measures, such as warm saline or vasodilators, should be implemented (Serin et al., 2022).

This study has several limitations. Firstly, the diagnosis of flap crisis was based on clinical observation and the lack of objective quantification may introduce potential diagnostic variability. Secondly, data on several important confounders, such as intraoperative vasopressor use, specific microsurgical techniques, and surgeon experience, were not available for analysis. Finally, while a standardized postoperative protocol was followed, the specific frequency of Doppler assessments and criteria for anticoagulation management were not explicitly detailed.

### Conclusions

Free flap transplantation has been proven to be a reliable approach for reconstructing oral and maxillofacial defects, while during of surgery and intraoperative blood loss are associated with flap crisis. The majority of flap crises occurred within the first 84 h postoperatively. Flap failure contributes to a significant increase in both the duration of postoperative hospital stay and the overall burden of medical cost.

## Supplemental Information

10.7717/peerj.21245/supp-1 Supplemental Information 1 Logistic regression analysis to identify independent risk factor for flap crisis

10.7717/peerj.21245/supp-2 Supplemental Information 2 Generalized Linear Mixed Model analysis to identify risk factor for flap crisis

10.7717/peerj.21245/supp-3 Supplemental Information 3 Correlation between flap crisis and flap failure based on different time

10.7717/peerj.21245/supp-4 Supplemental Information 4 Flap crisis probability

10.7717/peerj.21245/supp-5 Supplemental Information 5 STROBE checklist

10.7717/peerj.21245/supp-6 Supplemental Information 6 Raw data

10.7717/peerj.21245/supp-7 Supplemental Information 7 Codebook for the raw data

## Additional Information and Declarations

### Data Availability

The following information was supplied regarding data availability: The raw data is available in the Supplemental File.

## References

[ref-1] Chang EI, Chang EI, Soto-Miranda MA, Zhang H, Nosrati N, Crosby MA, Reece GP, Robb GL, Chang DW (2016). Comprehensive evaluation of risk factors and management of impending flap loss in 2,138 breast free flaps. Annals of Plastic Surgery.

[ref-2] Chen KT, Mardini S, Chuang DC, Lin CH, Cheng MH, Lin YT, Huang WC, Tsao CK, Wei FC (2007). Timing of presentation of the first signs of vascular compromise dictates the salvage outcome of free flap transfers. Plastic and Reconstructive Surgery.

[ref-3] Chorath K, Go B, Shinn JR, Mady LJ, Poonia S, Newman J, Cannady S, Revenaugh PC, Moreira A, Rajasekaran K (2021). Enhanced recovery after surgery for head and neck free flap reconstruction: a systematic review and meta-analysis. Oral Oncology.

[ref-4] Classen DA, Ward H (2006). Complications in a consecutive series of 250 free flap operations. Annals of Plastic Surgery.

[ref-5] Dawoud BES, Kent S, Tabbenor O, Markose G, Java K, Kyzas P (2022). Does anticoagulation improve outcomes of microvascular free flap reconstruction following head and neck surgery: a systematic review and meta-analysis. British Journal of Oral and Maxillofacial Surgery.

[ref-6] Devine JC, Potter LA, Magennis P, Brown JS, Vaughan ED (2001). Flap monitoring after head and neck reconstruction: evaluating an observation protocol. Journal of Wound Care.

[ref-7] Du W, Wu PF, Qing LM, Wang CY, Liang JY, Yu F, Tang JY (2015). Systemic and flap inflammatory response associates with thrombosis in flap venous crisis. Inflammation.

[ref-8] Eskander A, Kang S, Tweel B, Sitapara J, Old M, Ozer E, Agrawal A, Carrau R, Rocco JW, Teknos TN (2018). Predictors of complications in patients receiving head and neck free flap reconstructive procedures. Otolaryngology—Head and Neck Surgery.

[ref-9] Granzow J, Li AI, Caton A, Boyd JB (2015). Free flap survival following failure of the vascular pedicle. Annals of Plastic Surgery.

[ref-10] Harashina T (1988). Analysis of 200 free flaps. British Journal of Plastic Surgery.

[ref-11] Hekner DD, Roeling TA, Van Cann EM (2016). Perforator anatomy of the radial forearm free flap *versus* the ulnar forearm free flap for head and neck reconstruction. International Journal of Oral and Maxillofacial Surgery.

[ref-12] Irons GB, Wood MB, Schmitt 3rd EH (1987). Experience with one hundred consecutive free flaps. Annals of Plastic Surgery.

[ref-13] Largo RD, Selber JC, Garvey PB, Chang EI, Hanasono MM, Yu P, Butler CE, Baumann DP (2018). Outcome analysis of free flap salvage in outpatients presenting with microvascular compromise. Plastic and Reconstructive Surgery.

[ref-14] Megias Barrera J, Gorina Faz M, Artajona Garcia M, Costa Garcia A, Navarrete Pinero M (2019). Implementation of an easy-to-follow standardized chart for free flaps clinical monitoring during the start of a head and neck microsurgery unit in a tertiary hospital. Journal of Stomatology Oral and Maxillofacial Surgery.

[ref-15] Nakatsuka T, Harii K, Asato H, Takushima A, Ebihara S, Kimata Y, Yamada A, Ueda K, Ichioka S (2003). Analytic review of 2372 free flap transfers for head and neck reconstruction following cancer resection. Journal of Reconstructive Microsurgery.

[ref-16] Persson K, Torén M, Walther Sturesson L, Kander T, Nilsson C, Sjövall J (2025). Perioperative risk prediction in head and neck free flap reconstructive surgery. Acta Anaesthesiologica Scandinavica.

[ref-17] Ranganath K, Jalisi SM, Naples JG, Gomez ED (2022). Comparing outcomes of radial forearm free flaps and anterolateral thigh free flaps in oral cavity reconstruction: a systematic review and meta-analysis. Oral Oncology.

[ref-18] Russell J, Pateman K, Batstone M (2021). Donor site morbidity of composite free flaps in head and neck surgery: a systematic review of the prospective literature. International Journal of Oral and Maxillofacial Surgery.

[ref-19] Serin M, Bayramicli M, Cilingir Kaya OT, Levent HN, Akdeniz Dogan ZD, Ercan A, Kurt Yazar S (2022). The efficacy of hydrodilatation for the prevention of vasospasm following microsurgical anastomosis. Journal of Reconstructive Microsurgery.

[ref-20] Shen AY, Lonie S, Lim K, Farthing H, Hunter-Smith DJ, Rozen WM (2021). Free flap monitoring, salvage, and failure timing: a systematic review. Journal of Reconstructive Microsurgery.

[ref-21] Tsai TM, Bennett DL, Pederson WC, Matiko J (1988). Complications and vascular salvage of free-tissue transfers to the extremities. Plastic and Reconstructive Surgery.

[ref-22] Walia A, Lee JJ, Jackson RS, Hardi AC, Bollig CA, Graboyes EM, Zenga J, Puram SV, Pipkorn P (2022). Management of flap failure after head and neck reconstruction: a systematic review and meta-analysis. Otolaryngology—Head and Neck Surgery.

[ref-23] Wang W, Ong A, Vincent AG, Shokri T, Scott B, Ducic Y (2020). Flap failure and salvage in head and neck reconstruction. Seminars in Plastic Surgery.

[ref-24] Xie R, Liu Q, Zhang Y, Guo C, Huang X, Liu M (2023a). A wireless infrared thermometry device for postoperative flap monitoring: proof of concept in a porcine flap model. International Wound Journal.

[ref-25] Xie R, Zhang Y, Liu Q, Huang X, Liu M (2023b). A wireless infrared thermometry device for postoperative flap monitoring: proof of concept in patients. Surgical Innovation.

[ref-26] Yang X, Li S, Wu K, Hu L, Liu W, Ji T, Hu Y, Xu L, Sun J, Zhang Z, Zhang C (2016). Surgical exploration of 71 free flaps in crisis following head and neck reconstruction. International Journal of Oral and Maxillofacial Surgery.

