# Supplementary material for: Clinical characteristics of free flaps for oral and maxillofacial reconstruction: a retrospective study of 700 flaps over 3 years
Source: PeerJ. 2026 Jun 9;14:e21245. doi: 10.7717/peerj.21245 (PMC13262541; doi:10.7717/peerj.21245)
Supplement: Supplemental Information 1 [file peerj-14-21245-s001.docx]

| Logistic regression analysis to identify independent risk factor for flap crisis |
| --- |

| Variables | |  | p-value | OR (95%CI) |  |
| --- | --- | --- | --- | --- | --- |
|  | Intraoperative blood loss (ml) | 0.153 | 1.000 | (1.000-1.001) |  |
|  | BMI（kg / m²） | 0.067 | 0.927 | (0.854-1.005) |  |
|  | Duration of surgery(hours) | 0.019 | 1.167 | (1.025-1.329) |  |
|  | poor lifestyle habits | 0.268 | 0.743 | (0.440-1.256) |  |
|  | Prior chemoradiotherapy | 0.255 | 0.622 | (0.275-1.407) |  |
|  | DM | 0.240 | 0.630 | (0.292-1.361) |  |
|  | hypertension | 0.112 | 0.593 | (0.311-1.130) |  |
