# Supplementary material for: Clinical characteristics of free flaps for oral and maxillofacial reconstruction: a retrospective study of 700 flaps over 3 years
Source: PeerJ. 2026 Jun 9;14:e21245. doi: 10.7717/peerj.21245 (PMC13262541; doi:10.7717/peerj.21245)
Supplement: Supplemental Information 2 [file peerj-14-21245-s002.docx]

Generalized Linear Mixed Model analysis to identify risk factor for flap crisis

| Variables | *P*- value |
| --- | --- |
| Flap count | 0.000 |
| Intraoperative blood loss (ml) | 0.092 |
| BMI（kg / m²） | 0.113 |
| During of surgery(hours) | 0.078 |
| poor lifestyle habits | 0.492 |
| Prior chemoradiotherapy | 0.279 |
| DM | 0.512 |
| hypertension | 0.267 |
