# Supplementary figures and images for: Clinical characteristics of free flaps for oral and maxillofacial reconstruction: a retrospective study of 700 flaps over 3 years
Source: PeerJ. 2026 Jun 9;14:e21245. doi: 10.7717/peerj.21245 (PMC13262541; doi:10.7717/peerj.21245)

# Time distributinon of type of flap cirsis and flap failure

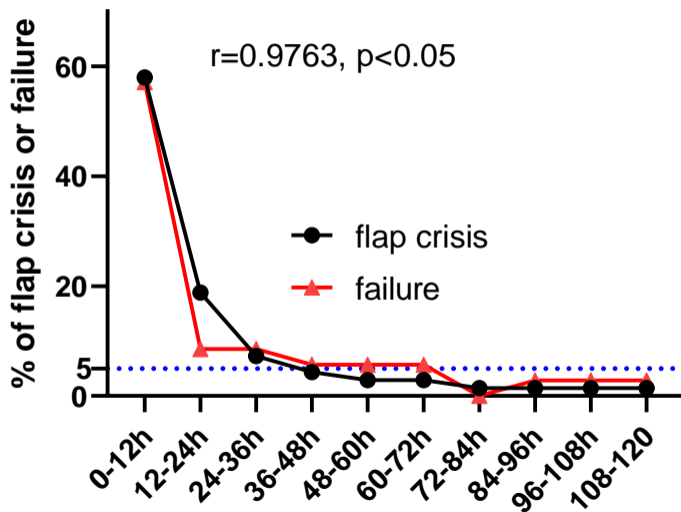

Supplement: Supplemental Information 3 [file peerj-14-21245-s003.pdf]

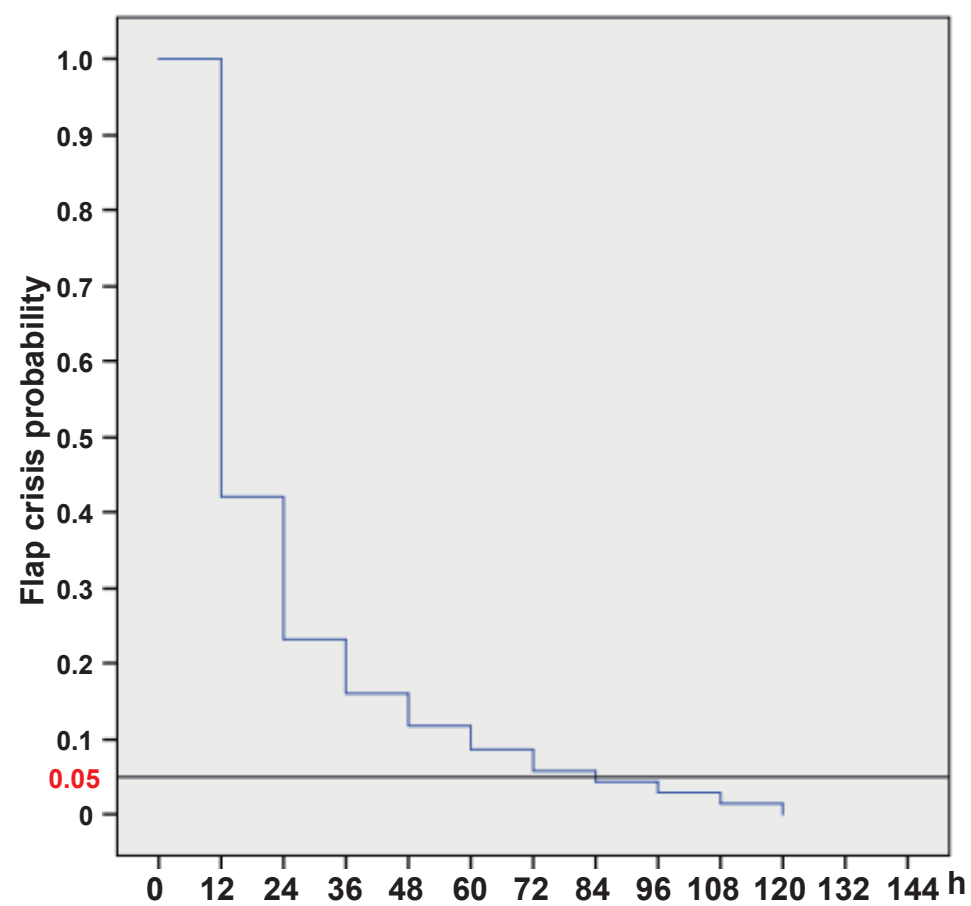

Supplement: Supplemental Information 4 [file peerj-14-21245-s004.pdf]
