# Supplementary material for: Clinical characteristics of free flaps for oral and maxillofacial reconstruction: a retrospective study of 700 flaps over 3 years
Source: PeerJ. 2026 Jun 9;14:e21245. doi: 10.7717/peerj.21245 (PMC13262541; doi:10.7717/peerj.21245)
Supplement: Supplemental Information 5 [file peerj-14-21245-s005.docx]

STROBE Statement—checklist of items that should be included in reports of observational studies

|  | Item No. | Recommendation | Page  No. | Relevant text from manuscript |
| --- | --- | --- | --- | --- |
| **Title and abstract** | 1 | (*a*) Indicate the study’s design with a commonly used term in the title or the abstract | Page1, lines 1-2 | Clinical characteristics of free flaps for oral and maxillofacial reconstruction: A Retrospective Study of 700 Flaps Over 3 Years |
|  |  | (*b*) Provide in the abstract an informative and balanced summary of what was done and what was found | Page 2, lines 21-40 | **Objective:** This study investigated the clinical characteristics and management of free flaps for oral and maxillofacial reconstruction.  **Methods:** A total of 689 subjects were categorized into the no-flap crisis group (620 flaps in 620 subjects) and the flap crisis group (80 flaps in 69 subjects). Demographic and clinical data, including gender, age, disease types, body mass index, preoperative chemoradiotherapy, poor lifestyle habits, postoperative length of stay, medical cost, intraoperative blood loss, during of surgery, hypertension and diabetes mellitus, were analyzed. In the flap crisis group, the distribution characteristics of flap types and the timing of crisis were examined.  **Results:** The overall survival rate of free flaps was 95 % (95% CI: 93.4 – 96.6), with a salvage rate of 49.28% (95% CI: 37.2 – 61.4). No significant differences were found between the two groups in terms of gender, age, body mass index, preoperative chemoradiotherapy, hypertension, diabetes mellitus or poor lifestyle habits. However, significant differences were observed in postoperative length of stay, duration of surgery, medical cost and intraoperative blood loss. In the flap crisis group, 58 flaps underwent crises within the first 36 hours, and a strong correlation was found between flap crisis and flap failure.  **Conclusion:** These findings indicate the reliability of free flap transplantation. Flap crises required prompt intervention, and a subsequent surgery should be considered when irreversible flap crisis is suspected. |
| Introduction | | | |  |
| Background/rationale | 2 | Explain the scientific background and rationale for the investigation being reported | Page 3-4, lines 45-68 | Over the past few decades, free flap transfer has become a cornerstone of oral and maxillofacial reconstruction. The survival rate of these flaps is generally considered to be in the region of 95%. Free flaps cover defects and restore partial functions in the oral and maxillofacial regions, improving swallowing and facial appearance. Successful transplantation not only boosts the confidence of both doctors and patients but also helps reduce medical costs. However, flap failure may lead to functional and cosmetic impairments, additional surgery, prolonged hospitalization, increased medical expenses, and delays in adjuvant therapy.  Close postoperative monitor is essential for assessing the perfusion of free flaps. Critical parameters include flap color, temperature, capillary refill, turgor, and bleeding characteristics. Compromised perfusion triggers progressive changes in these indicators. Without timely intervention, the transplanted flap is at risk of failure. Currently, the optimal time window for treating flap crises remains unclear. Animal models of venous congestion show changes in the color, texture, and histology of skin flaps over time suggesting that the time window for thrombogenesis is approximately 4 hours. However, such findings may not directly translate to clinical practice. Even when a flap crisis is suspected, there is no consensus regarding the most effective approach, whether observation, drug treatment or reoperation.  Furthermore, the characteristics of flap types and flap crises require further research. Although various methods exist to manage irreversible flap compromise, many aspects of these strategies still need further optimization and clarification. |
| Objectives | 3 | State specific objectives, including any prespecified hypotheses | Page 4, lines 69-70 | In this study, the clinical characteristics of free flaps used in oral and maxillofacial reconstruction during the past 3 years were analyzed. |
| Methods | | | |  |
| Study design | 4 | Present key elements of study design early in the paper | Page 4-5, lines 73-111 | Retrospective cohort study. To evaluate postoperative flap perfusion outcomes and risk factors in patients undergoing free flap reconstruction for oral and maxillofacial defects. |
| Setting | 5 | Describe the setting, locations, and relevant dates, including periods of recruitment, exposure, follow-up, and data collection | Page 5-6  Lines 89-111 | Postoperative flap perfusion was evaluated through bedside observation, focusing on key indicators such as flap color, temperature, capillary refill, turgor, and bleeding. Postoperative monitoring was performed following a tapering frequency schedule: every 30 minutes on day 1, hourly on day 2, and every 4 hours from days 3 to 7. Additionally, arterial flow was evaluated three times daily using a needle prick test. The criteria for identification of flap crisis and flap failure were as follows:  1. Venous crisis (VC): Appearance of dark spots or violet patches worsening within 30 minutes.  2. Arterial crisis (AC): Skin wrinkling and pallor, which was confirmed by Doppler ultrasound or subcutaneous puncture.  3. Mixed crisis (MC): A mixed pattern of vascular crisis exhibiting characteristics of both arterial and venous compromise.  4. Flap failure: Pale, bloodless puncture, wound dehiscence, odor, or infection.  5. All flap crises and failures were confirmed by two surgeons, each with more than five years of clinical experience. Completed necrosis of flap was defined as flap failure.  The subjects were categorized into the flap crisis group (80 flaps in 69 subjects) and the no flap crisis group (620 flaps in 620 subjects). The clinical and demographic information of subjects was recorded, including gender, age, body mass index (BMI), disease types, preoperative chemoradiotherapy, poor lifestyle habits (smoking or alcohol use), postoperative length of hospital stay, medical cost, intraoperative blood loss, during of surgery, hypertension, diabetes mellitus（DM） and the timing and type of flap crisis. |
| Participants | 6 | (*a*) *Cohort study*—Give the eligibility criteria, and the sources and methods of selection of participants. Describe methods of follow-up  *Case-control study*—Give the eligibility criteria, and the sources and methods of case ascertainment and control selection. Give the rationale for the choice of cases and controls  *Cross-sectional study*—Give the eligibility criteria, and the sources and methods of selection of participants | Page 4-5, lines 73-88 | A retrospective review was conducted on a consecutive series of 700 free flaps performed for oral and maxillofacial defects in 689 subjects at Guangxi Medical University, College of Stomatology in Nanning, China. The study included flaps and related data of subjects from the Department of Oral and Maxillofacial Surgery between December 15, 2020, and September 20, 2023.  The study was undertaken in accordance with the principles of the Declaration of Helsinki. Given the retrospective nature of the study, the need for informed consent was waived. The protocol was approved by the Ethics Committee of Stomatology Hospital Affiliated with Guangxi Medical University (Approval No: 2024003).  The inclusion criteria were: (1) subjects who suffered from tumors; (2) subjects who had undergone major resections or had defects in oral and maxillofacial regions; (3) subjects treated with free flap reconstruction including anterolateral thigh flap (ALTF), radial forearm free flap (RFFF), deep circumflex iliac artery free flap (DCIA) or fibula flap, and flap types were decided by the characteristics of recipient defects and donor sites; (4) All free flaps were performed by surgeons with more than 5 years of experience. |
|  |  | (*b*) *Cohort study*—For matched studies, give matching criteria and number of exposed and unexposed  *Case-control study*—For matched studies, give matching criteria and the number of controls per case |  |  |
| Variables | 7 | Clearly define all outcomes, exposures, predictors, potential confounders, and effect modifiers. Give diagnostic criteria, if applicable | Page 5, lines 107, 109, 110  Page 5, lines 108,110 | 1. **Quantitative Variables:**  Age, Body Mass Index (BMI), Intraoperative Blood Loss, postoperative Length of Hospital Stay, medical Cost,  Timing of Flap Crisis.  2. **Categorical Variables:**  (1) Demographic:  Sex: Male/Female.  Bad Living Habits: Smoking (yes/no), Alcohol use (yes/no).  (2) Clinical/Surgical:  Defect Type, Disease Location: Specific region of the tumor or defect, Preoperative Radiation Therapy: Received (yes/no), Prior Chemoradiation Therapy: History of treatment (yes/no). hypertension(yes/no)， diabetes mellitus(yes/no). Flap Type: ALTF, RFFF, DCIA, fibula flap.  (3) Outcome-Related:  Flap Crisis Type: Venous crisis, arterial crisis.  Flap Failure: Yes/no. |
| Data sources/ measurement | 8* | For each variable of interest, give sources of data and details of methods of assessment (measurement). Describe comparability of assessment methods if there is more than one group | *Page 5, line 89-104;* | 1.Clinical bedside observations (flap color, temperature, capillary refill, turgor, bleeding)*.*  *2. Medical history taking.*  *3．M measurement*  *4.* *Medical records.* |
| Bias | 9 | Describe any efforts to address potential sources of bias | N/A |  |
| Study size | 10 | Explain how the study size was arrived at | Page 4, lines 73-75 | a consecutive series of 700 free flaps |

Continued on next page

| Quantitative variables | 11 | Explain how quantitative variables were handled in the analyses. If applicable, describe which groupings were chosen and why | Page 5, 107, 109, 110 | Age, Body Mass Index (BMI),  Intraoperative Blood Loss,  Postoperative Length of Hospital Stay, Medical Cost. |
| --- | --- | --- | --- | --- |
| Statistical methods | 12 | (*a*) Describe all statistical methods, including those used to control for confounding | Page 6, lines 113-118 | The data were analyzed using descriptive statistics, including means and standard deviations. Chi-square test, t test, multivariable logistic regression analysis and generalized linear mixed model analysis were applied. Patient ID was included as a random effect, and flap count—accounting for both multiple and salvage flaps—was incorporated as a model parameter. Analyses were conducted using SPSS software (version 23). A p-value of < 0.05 was considered statistically significant. |
|  |  | (*b*) Describe any methods used to examine subgroups and interactions | N/A |  |
|  |  | (*c*) Explain how missing data were addressed | N/A |  |
|  |  | (*d*) *Cohort study*—If applicable, explain how loss to follow-up was addressed  *Case-control study*—If applicable, explain how matching of cases and controls was addressed  *Cross-sectional study*—If applicable, describe analytical methods taking account of sampling strategy | N/A |  |
|  |  | (*e*) Describe any sensitivity analyses | N/A |  |
| Results | | | | |
| Participants | 13* | (a) Report numbers of individuals at each stage of study—eg numbers potentially eligible, examined for eligibility, confirmed eligible, included in the study, completing follow-up, and analysed | Page 6, lines 120-121 | A total of 689 subjects underwent oral and maxillofacial reconstruction between December 15, 2020, and September 20, 2023, involving 700 free flaps. |
|  |  | (b) Give reasons for non-participation at each stage | N/A |  |
|  |  | (c) Consider use of a flow diagram | N/A |  |
| Descriptive data | 14* | (a) Give characteristics of study participants (eg demographic, clinical, social) and information on exposures and potential confounders | Table 2. Demographic of patients | Table 2. Demographic of patients |
|  |  | (b) Indicate number of participants with missing data for each variable of interest |  |  |
|  |  | (c) *Cohort study*—Summarise follow-up time (eg, average and total amount) |  |  |
| Outcome data | 15* | *Cohort study*—Report numbers of outcome events or summary measures over time | Page 6， lines 120-124 | The overall viability rate of the 700 flaps was 95% (95% CI: 93.4 – 96.6). The proportions of flap types were: ALTF 75.43%, Fibula 17.57%, RFFF 5.86%, and DCIA 1.14%. |
|  |  | *Case-control study—*Report numbers in each exposure category, or summary measures of exposure | N/A |  |
|  |  | *Cross-sectional study—*Report numbers of outcome events or summary measures | N/A |  |
| Main results | 16 | (*a*) Give unadjusted estimates and, if applicable, confounder-adjusted estimates and their precision (eg, 95% confidence interval). Make clear which confounders were adjusted for and why they were included | Page 6, lines 125-134  Page 7， lines 135-138  Page 7, lines 140-143  Page 7-8, lines 149-155  Page 7-8, lines 158-162  Page 8, lines 164-171 | The disease types in both groups included cancers of the tongue, gum, cheek, and floor of the mouth, as well as ameloblastoma, bony tumors, and mucoepidermoid carcinoma.The mean age in the no-flap crisis group was 52.31 years, while the mean age in the flap crisis group was 50.87 years (p = 0.398). Similarly, there were no statistically significant differences in the prevalence of poor lifestyle habits (213 vs. 28, p = 0.352) or history of prior chemoradiotherapy (53 vs. 8, p = 0.374). The no-flap crisis group had a higher prevalence of both diabetes mellitus (61 vs. 10, p =0.442) and hypertension (104 vs. 16, p =0.341) compared to the flap crisis group. The mean BMI was higher in the no-flap crisis group than in the flap crisis group (22.52 vs. 21.92; p = 0.170).  subjects experiencing flap crisis demonstrated significantly greater intraoperative blood loss (691.88 ml vs. 598.85 ml, p = 0.049), longer operative duration (7.98 hours vs. 7.33 hours, p = 0.007), and extended hospital stays (19.84 days vs. 13.82 days, p = 0.000) compared to those without crisis. Consequently, total medical costs were substantially higher in the crisis group (72,484.24 CNY vs. 51,271.48 CNY, p = 0.000).  Multivariable logistic regression analysis revealed statistically significant association between flap crisis and during of surgery after adjusting for potential confounders (p =0.019, Supplemental Table 1). Generalized linear mixed model analysis demonstrated that flap count exhibited a significant association with flap crisis  The incidence of flap crisis was highest within the first 12 hours, with 40 flaps (57.97%) occurring during this period. There were 13 flaps (18.84%) within 12 to 24 hours and 5 flaps (7.25%) within 24 to 36 hours. Overall, 58 flaps (84.06%) experienced flap crisis within the first 36 hours. Furthermore, 7 flaps (10.14%) had a crisis between 36 and 72 hours, and 4 flaps (5.80%) beyond 72 hours. Based on Kaplan-Meier analysis, the probability of flap crisis decreased over time, while the rate of flap crisis was below 5% at 84 hours after flap transplantation.  The incidence of flap failure within the first 12 hours was higher compared to other time intervals (Table 3 & Supplemental Figure 1), with 20 flaps (57.14%) occurring in this timeframe, followed by 3 flaps (8.57%) between 12 and 24 hours, and 3 flap (8.57%) between 24 and 36 hours. Within the first 36 hours, 26 flaps (74.29%) failed. When comparing flap types, the incidence of flap failure was higher in ALTF (4.92%), DCIA (12.5%), and Fibula (5.69%) compared to RFFF (2.44%)  Flap salvage rate was further analyzed based on the timing of exploration after onset of flap crisis (Table 4). The salvage rate within 4 hours was 54.17% (13/24), while from 4 to 10 hours, the salvage rate increased to 66.67% (8/12). A total of 16 flaps underwent delayed exploration, with only 4 surviving. In total, 35 flaps failed, and various treatment methods were employed for these failed flaps, including 11 free flaps, 4 adjacent flaps, and debridement (20 cases) (Table 5). Postoperative length of stay, intraoperative blood loss, and medical costs increased for subjects with failed flaps in flap crisis group |
|  |  | (*b*) Report category boundaries when continuous variables were categorized | N/A |  |
|  |  | (*c*) If relevant, consider translating estimates of relative risk into absolute risk for a meaningful time period | N/A |  |

Continued on next page

| Other analyses | 17 | Report other analyses done—eg analyses of subgroups and interactions, and sensitivity analyses | N/A |  |
| --- | --- | --- | --- | --- |
| Discussion | | | | |
| Key results | 18 | Summarise key results with reference to study objectives | Page 9, lines 188-191  Page 9-10, lines 193-198  Page10, lines 201-207 | The present study found that the overall rate of free flap survival was 95% (95% CI：93.4 – 96.6), while the flap crisis rate was 9.86% (69/700). With intensive care and proactive treatment, the salvage rate following exploration was 49.28% (95% CI: 37.2 – 61.4).  While multivariable logistic regression analysis revealed that the duration of surgery acted as an independent factor. Generalized linear mixed model analysis demonstrated that flap count exhibited a significant association with flap crisis  This study found that nearly 95% of flap crises occurred within the first 84 hours. During the initial 36 hours, 26 flaps failed (74.29%). The correlation between flap crisis and flap failure was strong (r=0.9763, p<0.05, Supplemental Figure 1). Therefore, reducing the incidence of flap crises can significantly decrease the flap failure rate. While previous studies have noted that flap crises and failures tend to occur early, this is the first study to describe their direct correlation. |
| Limitations | 19 | Discuss limitations of the study, taking into account sources of potential bias or imprecision. Discuss both direction and magnitude of any potential bias | Pages 13, lines 273-279 | This study has several limitations. Firsty, the diagnosis of flap crisis was based on clinical observation and the lack of objective quantification may introduce potential diagnostic variability. Secondly, data on several important confounders, such as intraoperative vasopressor use, specific microsurgical techniques, and surgeon experience, were not available for analysis. Finally, while a standardized postoperative protocol was followed, the specific frequency of Doppler assessments and criteria for anticoagulation management were not explicitly detailed. |
| Interpretation | 20 | Give a cautious overall interpretation of results considering objectives, limitations, multiplicity of analyses, results from similar studies, and other relevant evidence |  |  |
| Generalisability | 21 | Discuss the generalisability (external validity) of the study results | Page 13-14,  Lines 281-285 | Free flap transplantation has been proven to be a reliable approach for reconstructing oral and maxillofacial defects, while during of surgery and intraoperative blood loss are associated with flap crisis. The majority of flap crises occurred within the first 84 hours postoperatively. Flap failure contributes to a significant increase in both the duration of postoperative hospital stay and the overall burden of medical cost |
| Other information | |  | | |
| Funding | 22 | Give the source of funding and the role of the funders for the present study and, if applicable, for the original study on which the present article is based | Page 14, line 289-291 | National Clinical Key Specialty Construction Project (Grant No. CZ000037）.  Guangxi Medical & Health Appropriate Technology Development and Promotion Application Project (S2024122). |

*Give information separately for cases and controls in case-control studies and, if applicable, for exposed and unexposed groups in cohort and cross-sectional studies.

**Note:** An Explanation and Elaboration article discusses each checklist item and gives methodological background and published examples of transparent reporting. The STROBE checklist is best used in conjunction with this article (freely available on the Web sites of PLoS Medicine at http://www.plosmedicine.org/, Annals of Internal Medicine at http://www.annals.org/, and Epidemiology at http://www.epidem.com/). Information on the STROBE Initiative is available at www.strobe-statement.org.
