# Supplementary material for: Clinical characteristics of free flaps for oral and maxillofacial reconstruction: a retrospective study of 700 flaps over 3 years
Source: PeerJ. 2026 Jun 9;14:e21245. doi: 10.7717/peerj.21245 (PMC13262541; doi:10.7717/peerj.21245)
Supplement: Supplemental Information 7 [file peerj-14-21245-s007.docx]

(1) crisis

0: no crisis

1: crisis

(2) poor lifestyle habits

0: no

1: yes

(3) Prior chemoradiotherapy

0: no

1: yes

(4) diabetes mellitus

0: no

1 :yes

(5) Hypertension

0: no

1: yes
